# Singlet oxygen model evaluation of interstitial photodynamic therapy with 5-aminolevulinic acid for malignant brain tumor

**DOI:** 10.1117/1.JBO.25.6.063803

**Published:** 2019-12-14

**Authors:** Atsuki Izumoto, Takahiro Nishimura, Hisanao Hazama, Naokado Ikeda, Yoshinaga Kajimoto, Kunio Awazu

**Affiliations:** a Osaka University, Graduate School of Engineering, Suita, Japan; b Osaka Medical College, Department of Neurosurgery, Takatsuki, Japan; c Osaka University, Graduate School of Frontier Biosciences, Suita, Japan; d Osaka University, Global Center for Medical Engineering and Informatics, Suita, Japan

**Keywords:** photodynamic therapy, singlet oxygen, 5-aminolevulinic acid, protoporphyrin IX, singlet oxygen model

## Abstract

Interstitial photodynamic therapy (iPDT) with 5-aminolevulinic acid (ALA) is a possible alternative treatment for malignant brain tumors. Further evaluation is, however, required before it can be clinically applied. Computational simulation of the photophysical process in ALA-iPDT can offer a quantitative tool for understanding treatment outcomes, which depend on various variables related to clinical treatment conditions. We propose a clinical simulation method of ALA-iPDT for malignant brain tumors using a singlet oxygen (O12) model and O12 threshold to induce cell death. In this method, the amount of O12 generated is calculated using a photosensitizer photobleaching coefficient and O12 quantum yield, which have been measured in several previous studies. Results of the simulation using clinical magnetic resonance imaging data show the need to specify the insertion positions of cylindrical light diffusers and the level of light fluence. Detailed analysis with a numerical brain tumor model demonstrates that ALA-iPDT treatment outcomes depend on combinations of photobleaching and threshold values. These results indicate that individual medical procedures, including pretreatment planning and treatment monitoring, will greatly benefit from simulation of ALA-iPDT outcomes.

## 1 Introduction

Photodynamic therapy (PDT), which involves light excitation of photosensitizers (PSs) to induce tissue destruction, has attracted attention as a potential alternative treatment for malignant brain tumors because of high tumor-to-normal (TN) tissue contrast, low photon energy, and minimal invasion.^1^^,^^2^ The treatment effectiveness of PDT has been demonstrated with Photofrin^3^, talaporfin sodium,^4^^,^^5^ and 5-aminolevulinic acid (ALA)-induced protoporphyrin IX (PpIX).^6^^,^^7^ ALA-induced PpIX is expected to have few side effects. Compared with other PSs, it has a higher TN uptake ratio^6^^,^^8^ and faster discharge from the body within 48 h.^9^ In addition, for deeply seated brain tumors, interstitial photodynamic therapy (iPDT) with ALA,^10^^,^^11^ which involves insertion of cylindrical light diffusers into tissues to efficiently direct photons to target regions, has been more effective in clinical studies compared with existing standard treatments.^12^^,^^13^ Several clinical studies have demonstrated that iPDT outcomes depend on the light dose, which is determined by the irradiation conditions, such as the number of inserted cylindrical light diffusers, their positions, the light fluence rate, and the irradiation time.^13^ The relationships between treatment outcomes and treatment conditions should be analyzed for safe and effective use of ALA iPDT. Although further clinical studies might be able to provide these data, the collection of malignant brain tumor cases is difficult because these cancers are rare. In addition, many technical difficulties remain to be overcome in directly measuring the interaction of PSs and light *in vivo*.^14^

A computational approach that uses numerical models of treatment procedures can provide a prospective methodology to analyze iPDT outcomes for various combinations of parameters related to treatment conditions without experimental measurements. Recently, such an approach, which is called “*in silico* clinical trial” or “computational clinical trial,” has been applied for regulatory evaluation in the development of drugs and medical devices.^15^^,^^16^ In evaluation of ALA-iPDT treatment outcomes for brain tumors, the computational approach does not require the collection of target patients and overcomes the difficulties of direct measurement of treatment outcomes. For a computational clinical trial of ALA-iPDT, a photophysical model based on the mechanism of ALA-iPDT action should be constructed to estimate treatment outcomes.

In ALA-iPDT, the major cytotoxic species generated by light excitation of the PS is singlet oxygen (O12).^17^ Such PDT is categorized as type II. For estimation of type II PDT outcomes, the amount of O12 generated is a promising metric^18^^–^^20^ compared with other previously proposed metrics, including light dose^21^^,^^22^ and PDT dose.^23^ Although direct measurement of O12 by near-infrared spectroscopy has been proposed for PDT monitoring,^24^ problems include a low signal-to-noise ratio and low spatial resolution, especially in iPDT. For the computational evaluation of iPDT outcomes, several studies have proposed O12 generation calculation methods using various photokinetic and photochemical parameters, including reaction rate parameters and oxygen supply rate parameters.^25^ Moreover, Zhu et al. proposed an empirical macroscopic O12 model to calculate the spatiotemporal accumulation of reacted O12.^14^^,^^26^ The parameters needed for the calculation were obtained from an *in-vivo* mouse experiment. However, for evaluation of human malignant brain tumor treatment, the various photokinetic and photochemical parameters for ALA-induced PpIX in human brain tissues need to be obtained.

In this study, to evaluate the ALA-iPDT outcomes *in silico*, we propose a method to calculate the accumulated concentration of the generated O12 with a simple model using a photobleaching coefficient and a singlet oxygen quantum yield (SOQY). For the proposed method, the necessary parameters, photobleaching coefficient and SOQY for ALA-induced PpIX, were previously measured in several studies.^27^^–^^32^ To show the feasibility of the application using clinical data, ALA-iPDT outcomes were calculated using magnetic resonance imaging (MRI) data from a patient with malignant brain tumors. To understand the ALA-iPDT outcomes, the spatial distribution of generated O12 and ALA-iPDT outcomes with respect to irradiation conditions were analyzed. The simulation can compensate for a lack of understanding of ALA-iPDT for effective treatment design and monitoring.

## 2 Theory and Method

### 2.1 Simulation Overview

**Fig. 1:**
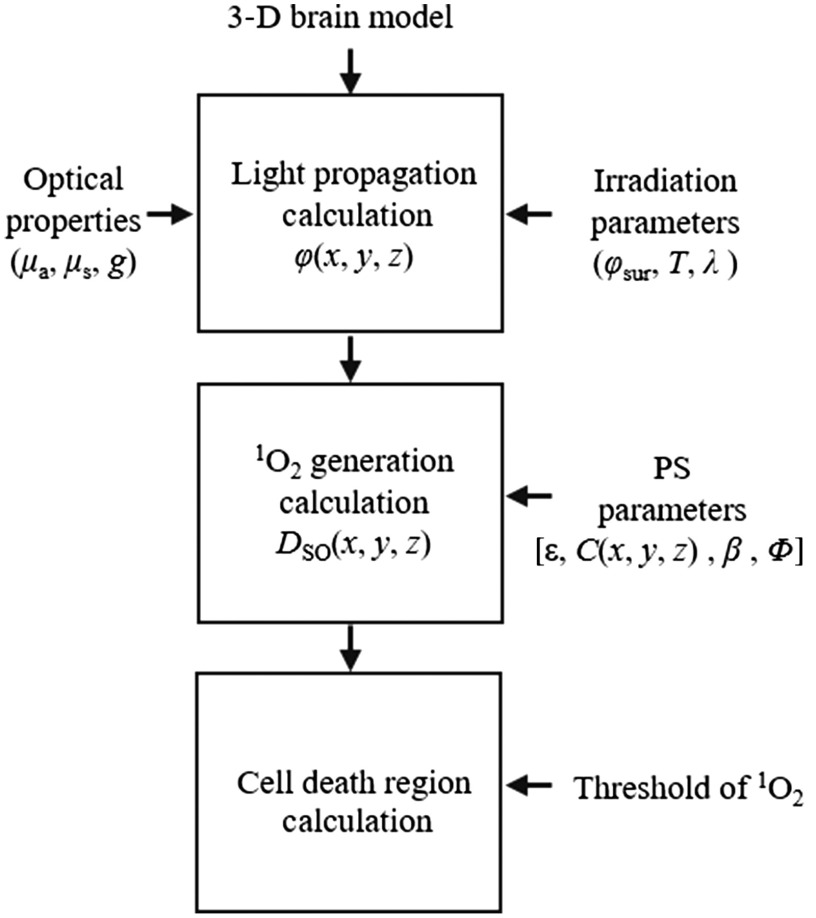
Schematic diagram of the calculation of ALA-iPDT outcomes. Here, $\mu_{a}$, $\mu_{s}$, and g are the absorption coefficient, scattering coefficient, and anisotropy of scattering, respectively; φ(x,y,z) is the spatial distribution of fluence rate at position (x,y,z); $\varphi_{\mathrm{sur}}$, T, and λ are the light fluences on the surfaces of inserted diffusers, irradiation time, and wavelength of light, respectively; $D_{\mathrm{SO}}(x,y,z)$ is the spatial distribution of generated O12 at position (x,y,z); ε, C(x,y,z), β, and Φ are the molar extinction coefficient, initial PS concentration at position (x,y,z), photobleaching coefficient, and quantum yield for O12 generation, respectively.

The simulation procedure to estimate ALA-iPDT outcomes consists of three major calculations: insertion conditions for cylindrical light diffusers and calculation of spatial distribution of light fluence rate at positions (x,y,z), φ(x,y,z), calculation of spatial distribution of generated O12 concentration, $D_{\mathrm{SO}}(x,y,z)$, and estimation of cell death region, as shown in Fig. 1. A numerical three-dimensional (3-D) brain model with optical properties and PpIX concentrations was prepared. Cylindrical light diffusers were virtually inserted in the numerical 3-D brain model and the light propagation was calculated to obtain φ(x,y,z). Using φ(x,y,z), the $D_{\mathrm{SO}}(x,y,z)$ was calculated. By setting the threshold of the generated O12 concentration, $D_{\mathrm{SO}(\mathrm{th})}$, above the level at which cell death is induced, the treatment area in the numerical 3-D brain model was calculated. In this study, for quantitative comparison of several treatment conditions, treated volume (TV) and damaged volume (DV) values are defined as the volume of cell death region by O12 in tumor tissues and normal tissues. Also, tumor coverage (TC) is defined as the ratio of TV to total tumor volume.

### 2.2 Numerical Tissue Model

To simulate ALA-iPDT outcomes, an anonymized MRI data set consisting of 156 two-dimensional slices (with resolution 512×512  pixels, pixel size 468  μm, and slice thickness 1.25 mm) from a patient with a malignant brain tumor was used. The MRI slices were manually segmented into normal and tumor regions by a clinician using Synapse Vincent software (Fujifilm, Tokyo, Japan). The normal tissue regions around the tumor regions were assumed to be the white matter regions because most adult malignant brain tumors arise in white matter.^33^ From the segmented MRI slices, a numerical 3-D voxel model having the segmented information was constructed^34^ and optical property parameters were assigned to each voxel according to its tissue type. The absorption coefficient, $\mu_{a}$ (${\mathrm{cm}}^{-1}$), scattering coefficient, $\mu_{s}$ (${\mathrm{cm}}^{-1}$), and anisotropy factor of scattering, g, were set as $1.7\;{\mathrm{cm}}^{-1}$, $365\;{\mathrm{cm}}^{-1}$, and 0.9, respectively, for the tumor regions and $0.7\;{\mathrm{cm}}^{-1}$, $951\;{\mathrm{cm}}^{-1}$, and 0.9, respectively, for the white matter regions at the wavelength of 635 nm.^35^ For iPDT outcome estimations, each voxel was divided into thirds in a direction perpendicular to the slice plane to give an isotropic voxel size of $468\times 468\times 417\;{\mu m}^{3}$.

For detailed analysis of the influence of photobleaching and the initial PpIX concentration, a uniform brain tumor tissue model (512×512×512  pixels, $100\times 100\times 100\;{\mu m}^{3}$), which consisted of tumor only, was used. Although PpIX is induced heterogeneously in tissues, the PpIX distributions were assumed to be uniform in the tumor region of the model for simplicity. The $\mu_{a}$, $\mu_{s}$, and g were set as $1.7\;{\mathrm{cm}}^{-1}$, $365\;{\mathrm{cm}}^{-1}$, and 0.9, respectively, for the wavelength of 635 nm.^35^ The photobleaching coefficient and initial PpIX concentration values were varied in evaluation.

### 2.3 Light Propagation Calculation

To obtain φ(x,y,z) for the constructed numerical 3-D models, the Monte Carlo-based method, which is regarded as the gold standard to calculate light propagation in nonhomogeneous scattering media, such as biotissues, was used.^34^ Here, the cylindrical fiber diffuser was assumed to have a radiation section that uniformly emits photons. The outer diameter was 1.1 mm and the radiation length was 40 mm. To calculate light propagation from the inserted cylindrical light diffuser, a 3-D Monte Carlo program (mcxyz.c)^34^ was customized. The launch points of photon packets were located inside the radiation section of the inserted cylindrical light diffuser according to Saccomandi et al.^36^ As a laser source, a multiport laser system (ML 7710i, Modulight, Finland) with a wavelength of 635 nm and eight fiber ports was assumed.^37^ Calculations of light propagation were performed for $1\times 10^{7}\text{ photons}$. Irradiation time and irradiation power density were changed according to the simulation conditions.

### 2.4 Singlet Oxygen Generation Calculation

To calculate $D_{\mathrm{SO}}(x,y,z)$, we adopted the SOQY model. PDT works well at oxygenation levels over 1% dissolved oxygen saturation rate (DO), but the effect decreases rapidly at lower levels of oxygenation.^38^ The oxygen concentrations of tissues during PDT did not fall below 1% DO.^39^ The SOQYs of ALA-induced PpIX in aqueous solutions have been reported and are very similar in 1% and 20% DOs, where the 1% and 20% DOs are calculated as 13 and 260  μM, respectively.^30^ In the calculation, the same SOQY value were used for the normal and tumor regions.

Using the SOQY of PpIX, Φ, $D_{\mathrm{SO}}(x,y,z)$ can be expressed as

$$D_{\mathrm{SO}}(x,y,z)=\int_{0}^{T}f(x,y,z)\cdot C(x,y,z,t)\cdot \Phi dt,$$ (1)

where T is the irradiation time, f(x,y,z) is the number of photons absorbed by PpIX in a unit time per unit concentration, and C(x,y,z,t) is the PpIX concentration at position (x,y,z) at time t. From the calculated φ(x,y,z), $D_{\mathrm{SO}}(x,y,z)$ can be expressed as

$$D_{\mathrm{SO}}(x,y,z)=\int_{0}^{T}\frac{1000\cdot \varepsilon \cdot \ln(10)\cdot \phi (x,y,z)\cdot \lambda }{hcN_{A}}\cdot C_{0}(x,y,z)\cdot \exp[-\frac{\phi (x,y,z)}{\beta }t]\cdot \Phi dt\;[\mathrm{mM}],$$ (2)

where ε is the molar extinction coefficient of PpIX, λ is the excitation light wavelength, h is Planck’s constant, c is the speed of light, $N_{A}$ is Avogadro’s constant, $C_{0}(x,y,z)$ is the initial PpIX concentration at position (x,y,z) at time t, and β is the photobleaching coefficient. Fixed values of the parameters were $\varepsilon =5000\;({\mathrm{cm}}^{-1}M^{-1})$, λ=635  (nm), $h=6.626\times 10^{-34}\;(Js)$, $c=3.0\times 10^{10}\;(\mathrm{cm}/s)$, $N_{A}=6.0\times 10^{23}\;({\mathrm{mol}}^{-1})$, and Φ=0.77.^30^^,^^40^ Other parameters were varied according to the evaluation items.

### 2.5 Cell Death Region Calculation

In the O12 threshold models,^26^^,^^41^ cell death is induced at the region where $D_{\mathrm{SO}}(x,y,z)$ exceeds $D_{\mathrm{SO}(\mathrm{th})}$. Normal tissues were assumed to be more resistant to O12 toxicity than tumor tissues because of superoxide dismutase, which exerts an important protective function against oxygen toxicity.^42^ However, to avoid underestimation of O12 toxicity in normal tissues, the $D_{\mathrm{SO}(\mathrm{th})}$ values for normal tissue damage were set to be equal to tumor tissue values. For estimation of the treatment region, the $D_{\mathrm{SO}(\mathrm{th})}$ value of 0.56 mM was used.^26^

## 3 Results

### 3.1 Estimation of ALA-iPDT Outcomes Using Clinical Magnetic Resonance Imaging Data

**Fig. 2:**
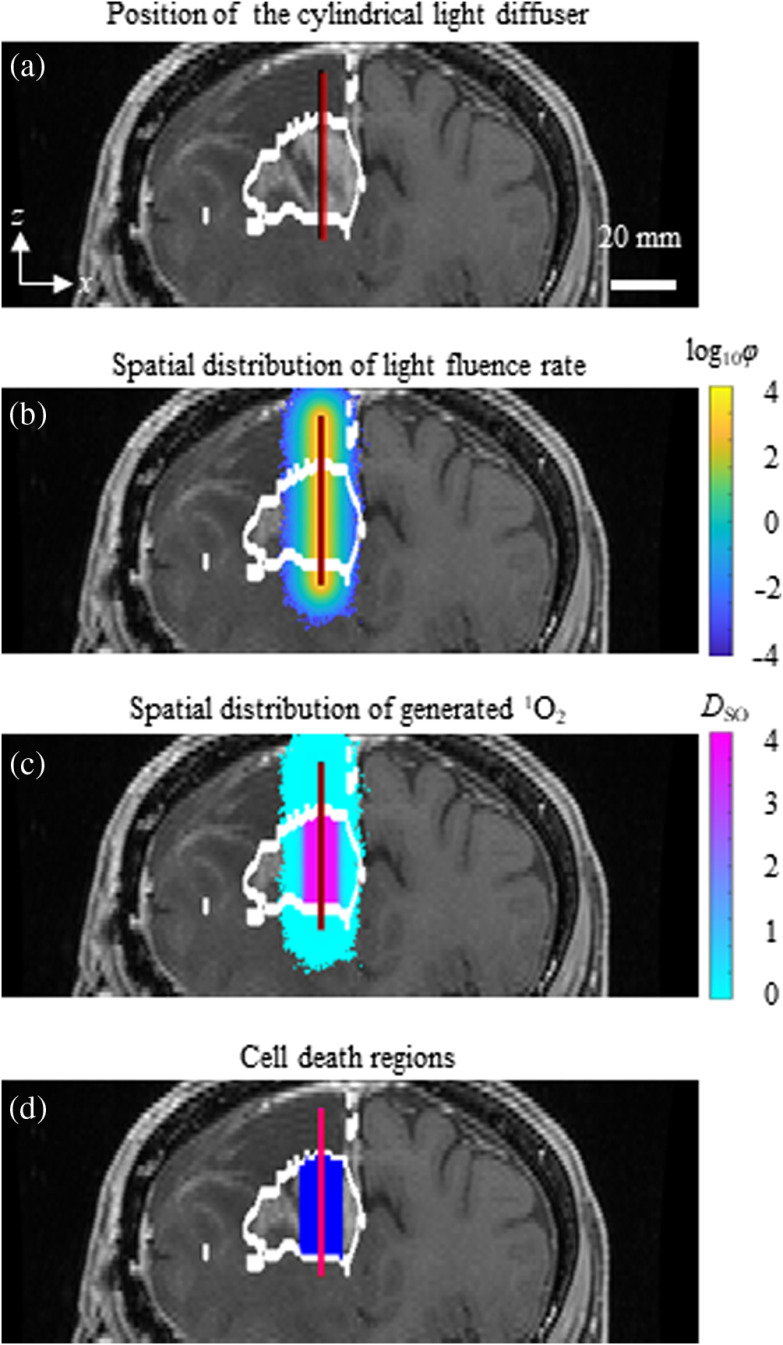
Calculation of treatment region under a set of irradiation treatment conditions. (a) Tumor regions segmented by a clinician and insertion position of a cylindrical light diffuser. Tumor regions are demarcated with white lines and the surface of the cylindrical light diffuser is shown in red. (b) Spatial distribution of light fluence, φ(x,y,z), calculated by a Monte Carlo-based algorithm, displayed as $\log_{10}[\varphi (x,y,z)]$ ($\mathrm{mW}/{\mathrm{cm}}^{2}$). (c) Spatial distribution of generated O12, $D_{\mathrm{SO}}(x,y,z)$ (mM), calculated from φ(x,y,z). (d) Estimated treatment region (indicated in blue) setting an O12 concentration threshold to induce cell death at 0.56 mM.

To estimate the iPDT outcomes using clinical data, the TV and DV values were calculated with a cylindrical light diffuser inserted as shown in Fig. 2. Figure 2(a) shows the segmented tumor region and the insertion position. The total volume of the tumor region was $30.8\;{\mathrm{cm}}^{3}$. According to clinical studies,^12^^,^^13^ T and the light fluence at the surface of the diffuser, $\varphi_{\mathrm{sur}}$, were set to 3600 s and $580\;{\mathrm{mW}/\mathrm{cm}}^{2}$ (linear density: 200  mW/cm), respectively. The $C_{0}(x,y,z)$ and $D_{\mathrm{SO}(\mathrm{th})}$ were set to 5.8  μM and 0.56 mM, respectively.^26^^,^^43^ Figures 2(b)–2(d) show the φ(x,y,z) calculated by the Monte Carlo-based method, the $D_{\mathrm{SO}}(x,y,z)$ calculated by inputting the calculated φ(x,y,z), and the treatment region where $D_{\mathrm{SO}}(x,y,z)$ exceeds $D_{\mathrm{SO}(\mathrm{th})}$. TV and DV were calculated as 2.48 and $0\;{\mathrm{cm}}^{3}$, respectively. No damaged tissue in the normal tissue region was found under these irradiation conditions. When the PpIX uptake ratio of the tumor tissue region to white matter tissue (TN ratio) was 95,^7^ the DV was $0\;{\mathrm{cm}}^{3}$ even when the initial PpIX concentration in the tumor region was the reported maximum value^43^ (28.2  μM). This result is consistent with a previous clinical study that showed almost no damage to white matter in ALA-PDT.^7^ However, when the TN ratio was set to 12, the value of the brain tissue adjacent to the tumor,^7^ damage in the white matter region occurred at higher PpIX initial concentrations (>10.6  μM) in the tumor region. This result shows that damage to normal tissue adjacent to the tumor is possible in clinical application.

### 3.2 Evaluation of Irradiation Conditions

**Fig. 3:**
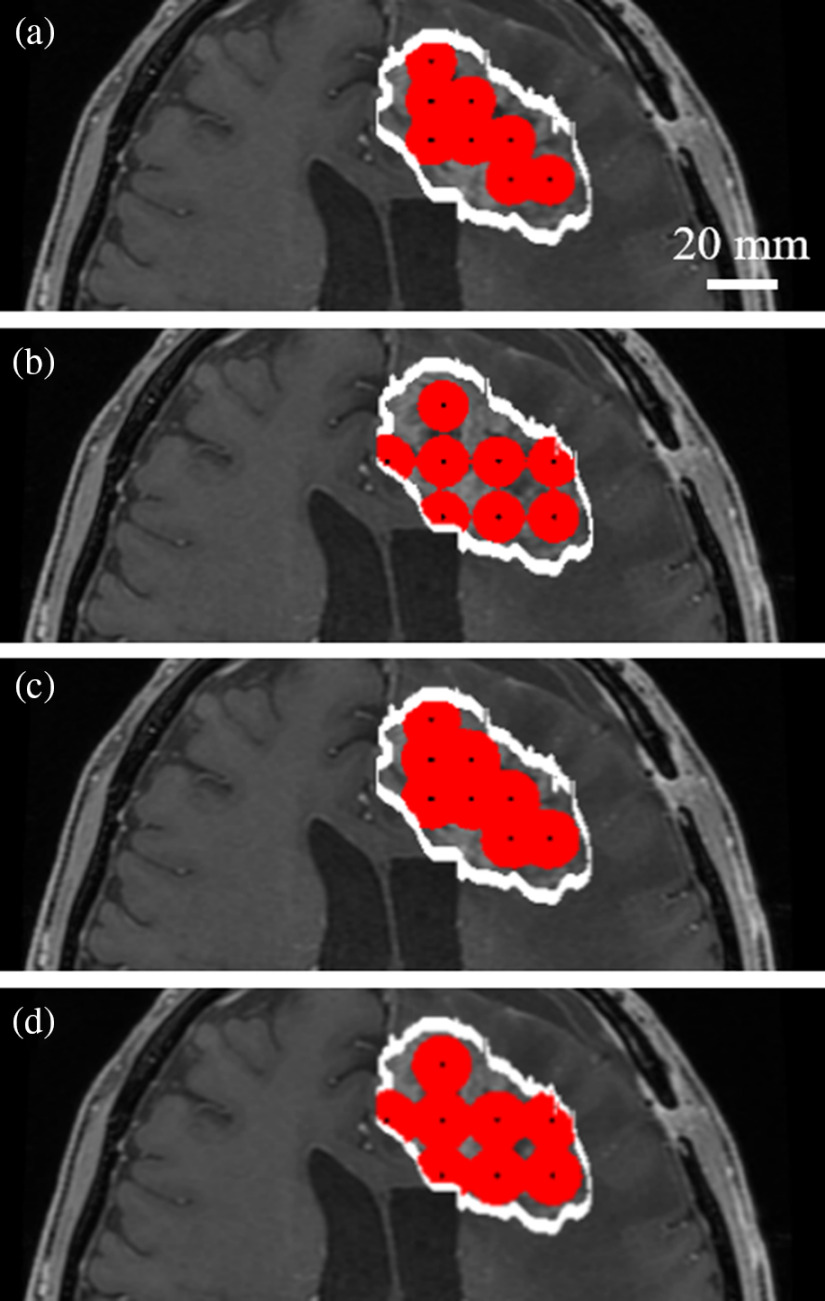
Estimated treatment regions with eight cylindrical light diffusers with $D_{\mathrm{SO}(\mathrm{th})}$ set to 0.56 mM. The tumor region is inside the white line. The treatment region is shown in red and the centers of the inserted fibers are indicated by black points. [Interdiffuser distance (cm), fluence rate at the diffuser surface ($\mathrm{mW}/{\mathrm{cm}}^{2}$)] = (a) (0.9, 580), (b) (1.25, 580), (c) (0.9, 2320), and (d) (1.25, 2320).

In ALA-iPDT, multiple cylindrical light diffusers are inserted depending on the tumor geometry.^12^ To evaluate the dependence of the ALA-iPDT outcome on the insertion positions and $\varphi_{\mathrm{sur}}$, TV and DV values were compared under several insertion conditions. The minimum distance between the diffusers (interfiber distance, L) has been determined as 0.9 cm to avoid a temperature increase above 42°C.^12^ The distance L was, therefore, chosen as 0.9 cm to concentrate photons between the diffusers as much as possible. Figure 3(a) shows the treated regions when L, T, and $\Phi_{\mathrm{sur}}$ were 0.9 cm, 1 h, and $580\;{\mathrm{mW}/\mathrm{cm}}^{2}$ (linear density: 200  mW/cm), respectively. TV and TC were calculated as $17.4\;{\mathrm{cm}}^{3}$ and 0.56, respectively. The treated regions surrounding each diffuser overlapped. To improve the TC by avoiding region overlap, L was increased. However, TC was calculated as 0.55 when L=1.25  cm [Fig. 3(b)]. Setting $\Phi_{\mathrm{sur}}$ as $2320\;{\mathrm{mW}/\mathrm{cm}}^{2}$ (linear density: 800  mW/cm), the TCs were 0.66 when L=0.9  cm [Fig. 3(c)] and 0.71 when L=1.25  cm [Fig. 3(d)]. The iPDT outcomes are strongly dependent on the relationship between insertion positions and $\Phi_{\mathrm{sur}}$. These results demonstrate that both the insertion position and the $\Phi_{\mathrm{sur}}$ should be designed by estimating the ALA-iPDT outcomes before treatment.

**Fig. 4:**
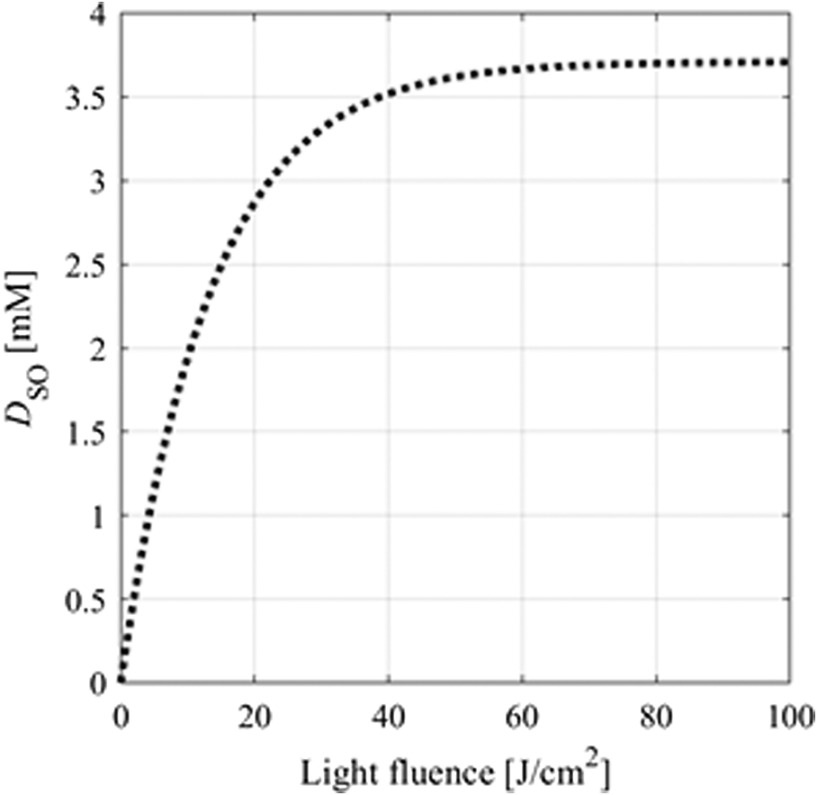
Relationship between generated O12 concentration, $D_{\mathrm{SO}}$, and the light fluence for 0 to $100\;{J/\mathrm{cm}}^{2}$.

The relationships between generated O12 concentration, $D_{\mathrm{SO}}$, and light fluence were investigated. As shown in Fig. 4, $D_{\mathrm{SO}}$ reaches a maximal value with increasing light fluence because of photobleaching. This indicates that an increase in light fluence does not always improve the ALA-iPDT outcomes. Moreover, in the O12-based models, $D_{\mathrm{SO}}$ is required to merely exceed the $D_{\mathrm{SO}(\mathrm{th})}$ value to provide treatment effects. When $D_{\mathrm{SO}(\mathrm{th})}$ was 0.4, 0.56, and 0.72 mM,^26^ the required light fluences were 1.6, 2.3, and $3\;{J/\mathrm{cm}}^{2}$, respectively. To avoid excessive irradiation, $D_{\mathrm{SO}(\mathrm{th})}$ should be considered when determining light fluence.

### 3.3 Analysis of Photobleaching Influence

**Fig. 5:**
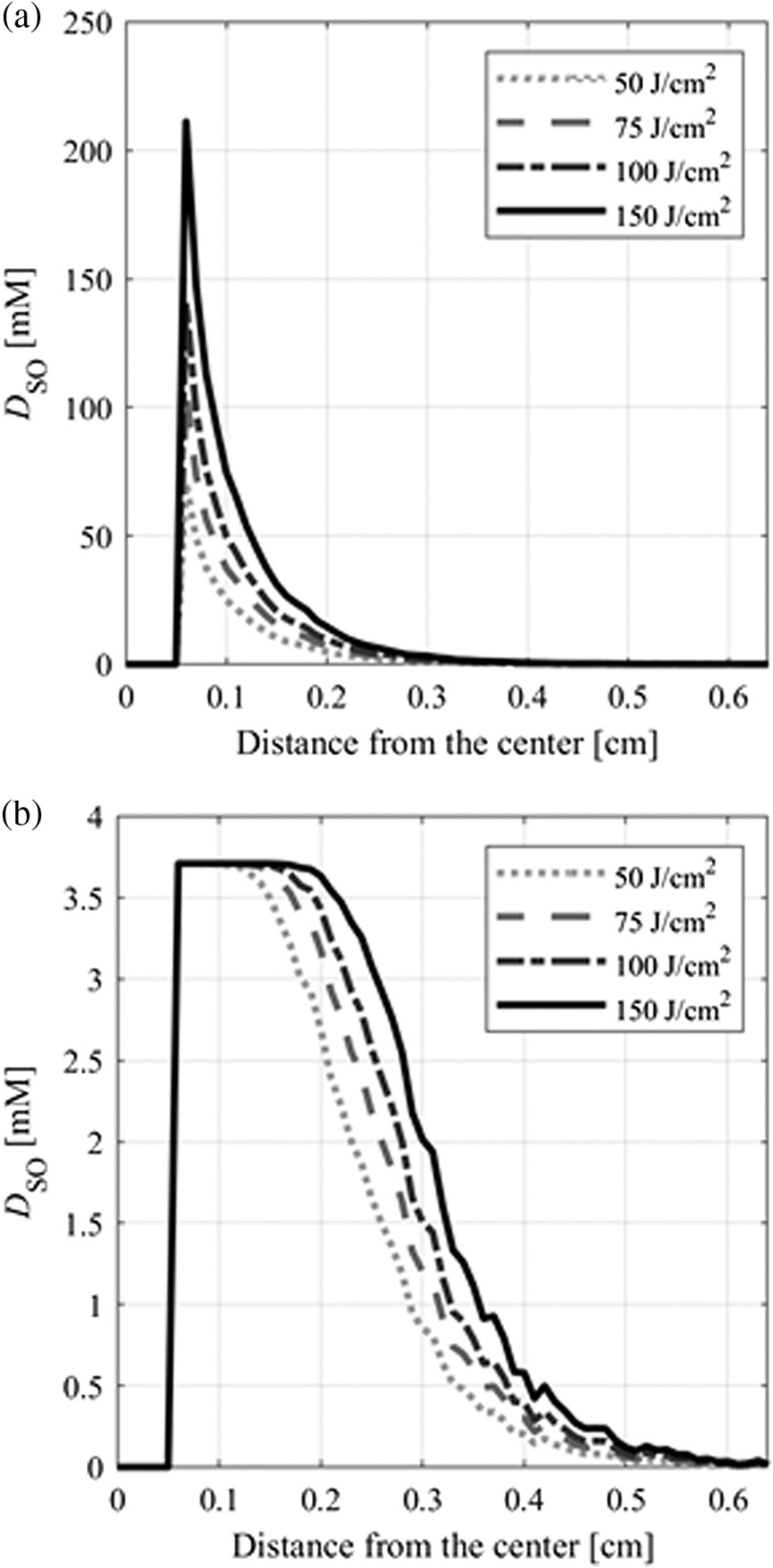
Profiles of generated O12 concentration, $D_{\mathrm{SO}}$, from the center of the inserted diffuser for light fluence at the surface of the diffuser of 50, 75, 100, and $150\;{J/\mathrm{cm}}^{2}$ (a) without and (b) with photobleaching when the coefficient was $13.5\;{J/\mathrm{cm}}^{2}$.

**Fig. 6:**
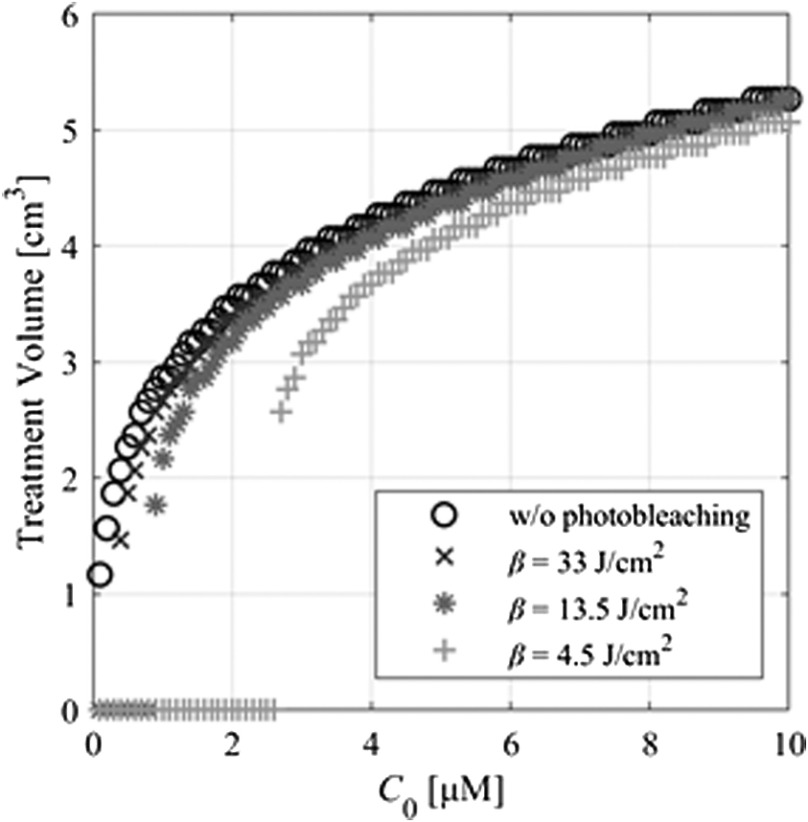
Relationship between treatment volume and PpIX initial concentration, $C_{0}$, of 0 to 10  μM in models with and without photobleaching for various values of photobleaching coefficients (4.5, 13.5, and $33\;{J/\mathrm{cm}}^{2}$).

To evaluate the photobleaching effect, $D_{\mathrm{SO}}$ was compared between the proposed model with and without the photobleaching process. The parameters β, $C_{0}$, which is PpIX initial concentration, T, and $\Phi_{\mathrm{sur}}$ were $13.5\;{J/\mathrm{cm}}^{2}$, 5.8  μM, 3600 s, and $580\;{\mathrm{mW}/\mathrm{cm}}^{2}$, respectively. Figure 5 shows $D_{\mathrm{SO}}$ with and without photobleaching. When $D_{\mathrm{SO}(\mathrm{th})}$ was assumed as 7.9 mM,^26^ the $D_{\mathrm{SO}}$ exceeded the threshold only in the model without photobleaching. When $D_{\mathrm{SO}(\mathrm{th})}$ was 0.4, 0.56, and 0.72 mM,^26^ the $D_{\mathrm{SO}}$ exceeded the threshold to induce cell death in both models. Therefore, the parameter combinations of photobleaching and $D_{\mathrm{SO}(\mathrm{th})}$ effect the iPDT outcomes. Next, using $C_{0}=5.8\;\mu M$, T=3,600  s, $\Phi_{\mathrm{sur}}=580\;{\mathrm{mW}/\mathrm{cm}}^{2}$, and $D_{\mathrm{SO}(\mathrm{th})}=0.56\;\mathrm{mM}$, TV was obtained to analyze the dependency of $C_{0}$, as shown in Fig. 6. When β was 4.5, 13.5, and $33\;{J/\mathrm{cm}}^{2}$,^27^^–^^29^ the ALA-iPDT outcomes were induced when each $C_{0}$ was more than 0.4, 0.9, and 2.7  μM. In the model without photobleaching, cell death was induced regardless of $C_{0}$. As β becomes smaller, more $C_{0}$ is necessary for treatment. The optical properties of malignant brain tumor vary from patient to patient.^35^ To estimate the possible ranges of the iPDT outcomes, TVs were calculated with the tissue optical properties, which maximized and minimized the light penetration depths. The maximum and minimum light penetration depths were defined using the standard deviation of the tissue optical properties. TV at the maximum light penetration depth was $20.0\;{\mathrm{cm}}^{3}$ when the $\mu_{a}$, $\mu_{s}$, and g were set as $0.92\;{\mathrm{cm}}^{-1}$, $113\;{\mathrm{cm}}^{-1}$, and 0.9, respectively. TV at the minimum light penetration depth was $2.1\;{\mathrm{cm}}^{3}$ when the $\mu_{a}$, $\mu_{s}$, and g were set as $2.5\;{\mathrm{cm}}^{-1}$, $617\;{\mathrm{cm}}^{-1}$, and 0.9, respectively. The TV difference was found since the reported standard deviations of optical properties are large.^35^ A detailed analysis of the optical properties of the brain normal and tumor tissues is expected to increase the precision of the iPDT outcome estimations.

### 3.4 Analysis of PS Parameter Sensitivities

**Fig. 7:**
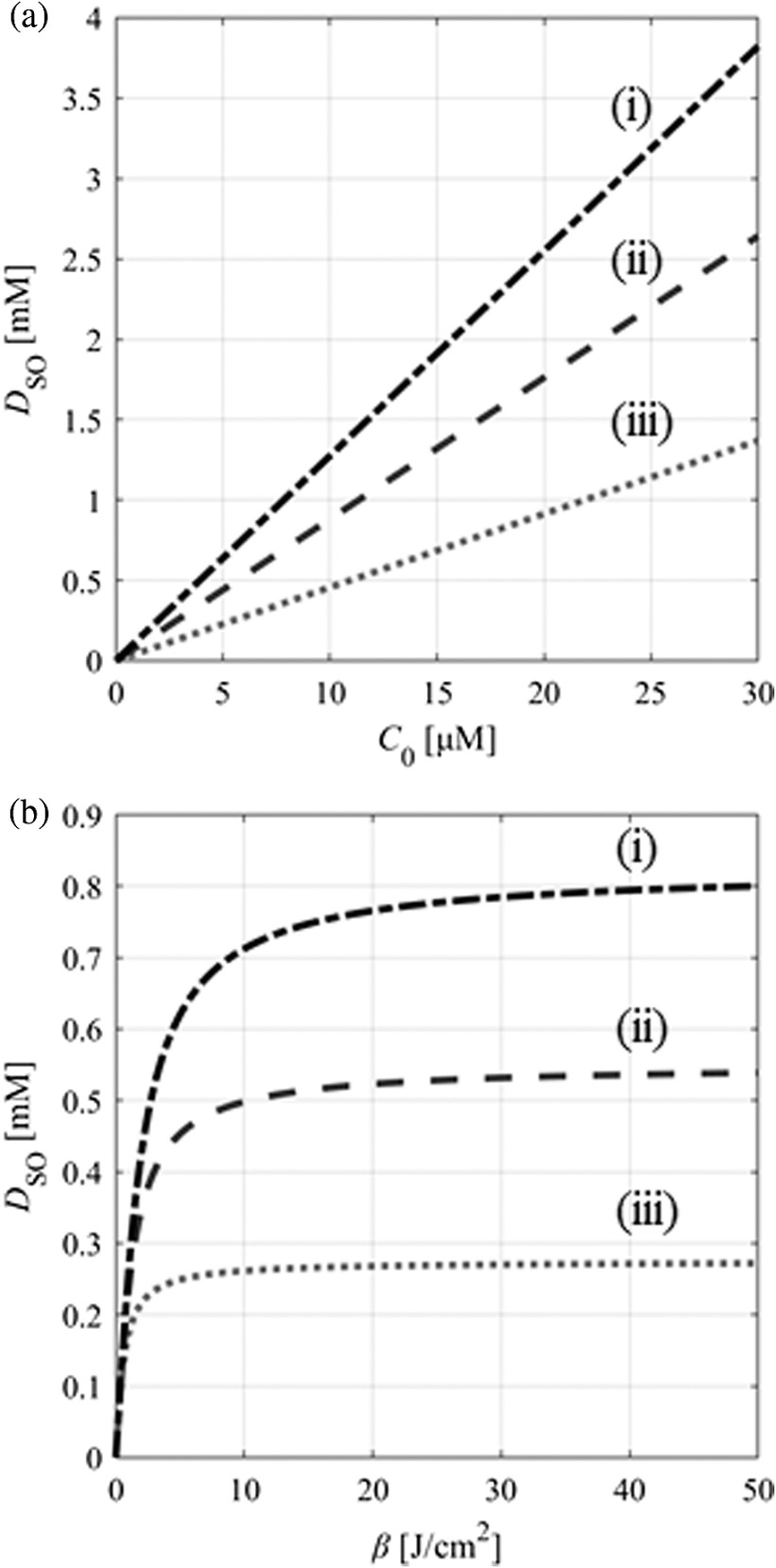
Generated O12 concentration, $D_{\mathrm{SO}}$, versus (a) PpIX initial concentration, $C_{0}$, from 0 to 30  μM ($\beta =13.5\;{J/\mathrm{cm}}^{2}$) and (b) photobleaching coefficient, β, of 0 to $50\;{J/\mathrm{cm}}^{2}$ ($C_{0}=5.8\;\mu M$). O12 concentration threshold, $D_{\mathrm{SO}(\mathrm{th})}$, was 0.56 mM and light fluences were (i) $3\;{J/\mathrm{cm}}^{2}$, (ii) $\text{2 }{J/\mathrm{cm}}^{2}$, and (iii) $\text{1 }{J/\mathrm{cm}}^{2}$.

The sensitivities of $C_{0}$ and β were evaluated using the uniform living tissue model with optical properties of malignant brain tumors. Figure 7(a) shows the $D_{\mathrm{SO}}$ when $C_{0}$ was varied, assuming β and $D_{\mathrm{SO}(\mathrm{th})}$ were $13.5\;{J/\mathrm{cm}}^{2}$ and 0.56 mM, respectively. When $C_{0}$ was above 13.8  μM, $1\;{J/\mathrm{cm}}^{2}$ light irradiation was enough to induce cell death. When $C_{0}$ was 6.32 to 13.8  μM, more light irradiation was necessary for treatment. Figure 7(b) shows the relationship between $D_{\mathrm{SO}}$ and β when $C_{0}=5.8\;\mu M$. The larger β led to higher $D_{\mathrm{SO}}$ because of a smaller decrease of C. When β was above $3.52\;{J/\mathrm{cm}}^{2}$, $3\;{J/\mathrm{cm}}^{2}$ light irradiation was enough for cell death. The $D_{\mathrm{SO}}$ varied depending on the light fluence and also on $C_{0}$ and β, which indicates that estimation of ALA-iPDT outcomes based only on light fluence, such as photon dose,^21^^,^^22^ is less accurate and that O12 dose should also be considered.

## 4 Discussion

For the qualitative evaluation of ALA-iPDT outcomes, an estimation method based on the single oxygen model was proposed and assessed using MRI data. Compared with other single oxygen models, the number of required parameters was small and their values were previously reported and were available for calculation. According to the previous paper,^39^ oxygen concentration was over 13  μM during PDT. The SOQY equals 0.77 in the concentration range.^30^ However, in the other paper,^18^ the lowest oxygen concentration during PDT was about 1  μM. At the oxygen concentration, the SOQY is calculated as 0.63.^30^ The SOQY is 18 % smaller compared to 0.77. It might affect the estimation accuracy of the ALA-iPDT. To prevent oxygen concentration decrease, oxygen supply control will offer a solution to keep the oxygen saturation in the brain tissues during PDT.^12^ To confirm whether the proposed model can explain the ALA-iPDT outcomes, we compared the simulated results with those from previously reported animal experiments.^7^ In the estimated result shown in Fig. 2, even under severe light irradiation conditions, normal tissue damage was not induced when the TN ratio was set to 92.^7^ This supports the findings of Lilge and Wilson^7^ who demonstrated that there was no white matter damage at this TN ratio in animal experiments. In the simulation with the maximum accumulated PpIX concentration (28.2  μM^43^), normal tissue damage occurred with a TN ratio less than 32. Therefore, for ALA-iPDT treatment, irradiation conditions should be decided by considering $C_{0}$ and the TN ratio to prevent normal tissue damage.

To investigate the influence of irradiation conditions on ALA-iPDT outcomes, TCs were evaluated according to the L and $\Phi_{\mathrm{sur}}$ values. As shown in Fig. 2, when $\Phi_{\mathrm{sur}}$ was $580\;{\mathrm{mW}/\mathrm{cm}}^{2}$ (200  mW/cm), the TC when L=0.9  cm was better than that when L=1.25  cm. L=1.25 was better for the $\Phi_{\mathrm{sur}}$ of $2320\;{\mathrm{mW}/\mathrm{cm}}^{2}$ (800  mW/cm). These results indicate that consideration of the relationship between L and $\Phi_{\mathrm{sur}}$ is required for pretreatment planning. The light fluence for effective treatment varied according to $D_{\mathrm{SO}(\mathrm{th})}$. Precise $D_{\mathrm{SO}(\mathrm{th})}$ will enable optimization of irradiation conditions and prevent excessive irradiation. $D_{\mathrm{SO}(\mathrm{th})}$ can differ depending on tumor and PS types;^25^ therefore, determination of $D_{\mathrm{SO}(\mathrm{th})}$ in ALA-iPDT for malignant brain tumor is required.

Photobleaching is required in the O12 model because photobleaching of ALA-induced PpIX has been observed in many studies.^27^^–^^29^ As shown in Fig. 6, when β of PpIX was 4.5, 13.5, or $33\;{J/\mathrm{cm}}^{2}$, cell death was induced when $C_{0}$ was more than 0.4, 0.9, and 2.7  μM, respectively. In the O12 model without photobleaching, cell death was induced regardless of $C_{0}$. In a clinical study of ALA-iPDT for malignant brain tumor^44^ and in an *in-vivo* study of ALA-PDT,^43^ an iPDT treatment effect was not confirmed when $C_{0}$ was 0.6 or below 1  μM, respectively. The estimated outcome was most consistent with the clinical and *in-vivo* results when $\beta =13.5\;{J/\mathrm{cm}}^{2}$. Parameters β and $C_{0}$, however, have wide variations because of many factors, including solution conditions and individual differences.^43^^,^^45^ Previously reported β and $C_{0}$ values for PpIX have ranges from 1.8 to $33\;{J/\mathrm{cm}}^{2}$ and from 0 to 28.2  μM, respectively.^43^^,^^45^ The difference in TV was $3.7\;{\mathrm{cm}}^{3}$ between cases when β was 4.5 and $33\;{J/\mathrm{cm}}^{2}$ ($C_{0}=5.8\;\mu M$). Our simulated results show that the PS parameters β and $C_{0}$ have significant influence on the estimation results. For precise monitoring of ALA-iPDT outcomes during treatment, β and $C_{0}$, which differ from patient to patient, should be measured *in situ*, for example by a fiber optic measurement.^46^ Use of the measured values leads to improvement of the estimation accuracy. However, since such parameter measurement before treatment is difficult, treatment planning will be performed by considering possible ranges of iPDT outcomes, which is derived from the reported parameter variances.^43^^,^^45^

By comparing the reported clinical and animal studies, the proposed method can explain the ALA-iPDT outcomes using measured parameters. In the computational evaluation, combinations of treatment conditions that are difficult to quantify in clinical studies can be evaluated. Therefore, the outcome estimation method will provide prospective applications for ALA-iPDT development. For example, it is capable of helping treatment design before treatment and treatment monitoring during treatment at a low cost in various treatment conditions. In addition, it is useful for low-cost evaluation of treatment conditions for rare diseases with few cases compared to actual clinical trials. The O12 model-based simulation can estimate the immediate outcomes after light irradiation. The immune response has also been reported as a treatment factor in ALA-PDT.^13^ Incorporation of the immune response in the simulation will further refine the estimation of ALA-iPDT outcomes.

## 5 Conclusions

Treatment outcomes of ALA-PDT for malignant brain tumors depend on several parameters, including tumor tissue geometry, diffuser geometry, light irradiation-related variables, PS-related variables, and O12 thresholds. In this study, to evaluate the ALA-PDT treatment conditions for malignant brain tumors, a treatment outcome estimation method was developed based on the mathematical model of O12 generation using the SOQY and photobleaching coefficient. The estimated outcome results using measured parameters are consistent with previously reported clinical and animal ALA-PDT studies. Comparison between the proposed model with and without photobleaching shows that the light dose model is inadequate for the estimation of ALA-iPDT outcomes. These results indicate that pretreatment planning and treatment monitoring require accurate $D_{\mathrm{SO}(\mathrm{th})}$, $C_{0}$, and β values for the simulation of ALA-PDT outcome. The precise measurement of these values will improve the accuracy and provide better treatment design and outcomes. The simulation can estimate the immediate outcomes by O12 after iPDT. The immune response is also considered as a treatment factor in ALA-PDT;^44^ therefore, incorporation of the immune response in the simulation will improve iPDT outcomes.
